# Genome Wide Association Mapping for Arabinoxylan Content in a Collection of Tetraploid Wheats

**DOI:** 10.1371/journal.pone.0132787

**Published:** 2015-07-15

**Authors:** Ilaria Marcotuli, Kelly Houston, Robbie Waugh, Geoffrey B. Fincher, Rachel A. Burton, Antonio Blanco, Agata Gadaleta

**Affiliations:** 1 Department of Soil, Plant and Food Sciences, Section of Genetics and Plant Breeding, University of Bari 'Aldo Moro', Via G. Amendola 165/A, Bari, Italy; 2 The James Hutton Institute, Invergowrie, Dundee, Scotland; 3 Australian Research Council Centre of Excellence in Plant Cell Walls, School of Agriculture, Food and Wine, University of Adelaide, Waite Campus, Glen Osmond, SA, Australia; Institute for Sustainable Agriculture (IAS-CSIC), SPAIN

## Abstract

**Background:**

Arabinoxylans (AXs) are major components of plant cell walls in bread wheat and are important in bread-making and starch extraction. Furthermore, arabinoxylans are components of soluble dietary fibre that has potential health-promoting effects in human nutrition. Despite their high value for human health, few studies have been carried out on the genetics of AX content in durum wheat.

**Results:**

The genetic variability of AX content was investigated in a set of 104 tetraploid wheat genotypes and regions attributable to AX content were identified through a genome wide association study (GWAS). The amount of arabinoxylan, expressed as percentage (w/w) of the dry weight of the kernel, ranged from 1.8% to 5.5% with a mean value of 4.0%. The GWAS revealed a total of 37 significant marker-trait associations (MTA), identifying 19 quantitative trait loci (QTL) associated with AX content. The highest number of MTAs was identified on chromosome 5A (seven), where three QTL regions were associated with AX content, while the lowest number of MTAs was detected on chromosomes 2B and 4B, where only one MTA identified a single locus. Conservation of synteny between SNP marker sequences and the annotated genes and proteins in *Brachypodium distachyon*, *Oryza sativa* and *Sorghum bicolor* allowed the identification of nine QTL coincident with candidate genes. These included a glycosyl hydrolase GH35, which encodes *Gal7* and a glucosyltransferase GT31 on chromosome 1A; a cluster of GT1 genes on chromosome 2B that includes *TaUGT1* and *cisZog1*; a glycosyl hydrolase that encodes a *CelC* gene on chromosome 3A; *Ugt12887* and *TaUGT1*genes on chromosome 5A; a (1,3)-β-D-glucan synthase (*Gsl12* gene) and a glucosyl hydrolase (*Cel8* gene) on chromosome 7A.

**Conclusions:**

This study identifies significant MTAs for the AX content in the grain of tetraploid wheat genotypes. We propose that these may be used for molecular breeding of durum wheat varieties with higher soluble fibre content.

## Introduction

Many clinical analysis, including a Food and Drug Administration (FDA) approved study [1], have demonstrated how dietary fibre from cereal grains, including arabinoxylans (AXs), are positively correlated with lower cholesterol levels and glycaemic index in humans.

Dietary fibre in cereal grains consists mainly of non-starchy polysaccharides (NSPs) of cell wall origin. These polysaccharides form solutions of high viscosity and their physiochemical and biological properties have beneficial physiological effects in the small and large intestine. The important properties of NSPs include their solubility in water, their propensity to form solutions of high viscosity, their ‘bulk’, and their fermentability to beneficial short chain fatty acids (SCFAs). When incorporated in the human diet these features may lead to a greatly diminished risk of coronary heart disease, colorectal cancer, inflammatory bowel disease, breast cancer, tumour formation, mineral related abnormalities and disordered laxation [2].

AXs are a major component of the cell walls of the endosperm in most cereal species. The structure consists of β-1,4 linked D-xylopyranosyl residues. Monomeric α-L-arabinofuranoside can be present at the *C(O)-3* and/or the *C(O)-2* position of the xylose moieties [3]. Rye and wheat have the highest contents, with reported ranges of 7.1–12.2 g/100g of whole grain and 4–9 g/100g of whole grain, respectively, followed by barley, maize, rice, and oats [2]. The lowest levels (<2%) are found in sorghum [2]. Wheat is one of the most important crops in the word for food production and has a relatively high level of AX [4,5]. Wheat therefore represents a potentially important route to improve the diet and health of large numbers of people, without the need for a change in human behaviour regarding food choices.

To date, much of the work that has been carried out to understand the genetics underlying AX content has been focused predominantly on hexaploid wheat. Mitchell et al. [6] identified a group of candidate genes for AX biosynthesis from several glycosyltransferase (GT) families, including, GT2, GT43, GT47, GT48, GT61, GT64, and GT77. Subsequent studies demonstrated that groups of GT61, GT43 and GT47 genes were associated with the synthesis of the xylan backbone and the addition of its substituents, which commonly include arabinosyl, glucuronyl and feruloyl residues [7]. Members of the GT61 family (*TaXAT*) are responsible for monosubstitution of the majority of the wheat starchy endosperm AX with α-(1,3)–linked arabinofuranose [6], while candidates from the GT43 and GT47 families, *TaGT43_2*, *TaGT47_2*, with all three homoeologues expressed in the starchy endosperm, believed to be involved in synthesis of the AX backbone [8].

In classical quantitative genetic studies, the construction of linkage maps in biparental populations has allowed the estimation of the number of loci controlling genetic variation in segregating populations, and has led to the characterization of loci with regard to gene action, phenotypic effects, pleiotropic effects and epistatic interactions with other QTL [9]. In hexaploid wheat, several studies have demonstrated the variability of AX content confirming the quantitative nature of this trait [10], which has been shown to vary from 4% to 9% [2] compared to 4% and 6% of the total grain weight in tetraploid wheat [11]. However, inheritance and environmentally correlated changes are not well defined, especially in durum wheat. Charmet et al. [12], using two recombinant populations, identified two QTL regions on chromosomes 1B and 6B. Nguyen et al. [13] used a cross between Berkut × Krichauff DH, hexaploid wheat cultivars, and reported QTL for total AX content on chromosomes 1A, 2A, 3D, 4D, 6B, and 7A. Two of these QTL, *QGax*.*aww-2A*.*1* and *QGax*.*aww-4D*.*1* (located on chromosomes 2A and 4D), were found to have a major effect on AX content in the endosperm. Quraishi et al. [14] identified QTL for AX on chromosomes 1B, 3A, 3D, 5B, 6B, 7A, 7B and meta-QTL on chromosomes 1B, 3D and 6B.

An alternative approach for QTL detection, commonly referred to as “association mapping” (AM) or genome-wide association study (GWAS), is based on the detection of correlations between genotype and phenotype in a group of individuals represented by a germplasm collection or natural populations [15]. This method exploits linkage disequilibrium (LD) and the higher amount of recombination typically available in a population used for AM compared to a biparental mapping population to identify regions of the genome associated with the trait of interest [15]. Compared to classical linkage analysis, which uses the recombination events within a group of individuals (i.e., old and new varieties, landraces, experimental material etc.), the GWAS detects non-random associations between phenotypes and genotypes. However, the population structure within the collection of individuals used for GWAS, as a result of admixture, mating system, genetic drift, and/or artificial or natural selection that occurred during evolution, domestication, or plant improvement can result in false positive correlations between markers and traits observed as an over inflation of p values [16]. The quality of the phenotypic data, population size, population structure, ascertainment bias [17] and degree of LD are the primary determinants for the success of AM [18].

Recently, a high-density wheat SNP iSelect array comprising approximately 90,000 gene-associated SNPs providing dense coverage of the wheat genome was developed by Wang et al. [17] and can be used to genotype wheat collections effectively allowing the detection of QTL. The objectives of the present work were the detection of genetic regions associated with AX content in a tetraploid wheat collection, characterized by SNP markers, and the identification of functionally relevant candidate genes for this trait.

## Materials and Methods

### Plant material

A core collection of 104 tetraploid wheat accessions was evaluated in replicated trials in southern Italy in 2012 in the experimental field of the University of Bari at Valenzano (Bari, Italy) (field studies did not involve endangered or protected species and for those no permission were required). This core collection, including 61 cultivars of durum wheat (ssp. *durum*), divided in three subgroup according on the date of release, 20 accessions of ssp. *turgidum*, and 8 of ssp. *turanicum*, 8 of ssp. *polonicum*, 8 of ssp. *carthlicum*, 9 of ssp. *dicoccum*, and 8 of ssp. *dicoccoides*, was constituted accounting the genetic variability of a wide collection characterized by Laidò et al. [19]. A randomized complete block design with three replications and plots consisting of 1m rows, 30 cm apart, with 80 germinating seeds per plot, was used in the field experiment. During the growing season, 10 g nitrogen per m^2^ was applied and standard cultivation practices were adopted. Plots were hand-harvested at maturity and grain was stored at 4°C until processed. The entire set of accessions was phenotypically characterized for total AX content by reverse phase HPLC (Agilent Technologies) following the method used by Burton et al. [20].

### Molecular markers

A total of 50 ng/μL of genomic DNA of each line plus Chinese Spring nulli-tetrasomic (NT) lines was analysed using the wheat 90K iSelect array [17]. Genotyping was performed with Trait Genetics GmbH (http://www.traitgenetics.de) following the manufacturer’s recommendations as described in Akhunov et al. [21]. The genotyping assays were carried out in an Illumina iScan reader and performed using GenomeStudio software version 2011.1 (Illumina). NT lines [22] of Chinese Spring were used to assign SNP markers to each chromosome as described in Colasuonno et al. [23]. Prior to GWAS, markers with a minimum allele frequency of less than 10% and those that had >5% missing data points were removed from the data matrix.

### Monosaccharide analysis

The total AX content for each line was measured on 20 mg whole grain flour by reverse phase HPLC (Agilent Technologies) as described by Burton et al. [20].

Samples were initially hydrolysed in 1 M sulphuric acid at 97°C for 3 hr. The hydrolysate was diluted (1:20) and derivatized with 1-phenyl-3-methyl-5-pyrazolone (PMP) at the anomeric carbon atoms. A Phenomenex Kinetex 2.6 μm C18 100 x 3 mm 100Å column at 30°C and a flow rate of 0.8 mL/min was used to perform the chromatography. The separation of PMP-monosaccharide derivatives was performed with an eluent containing 40 mM ammonium acetate in 10% acetonitrile (pH~6.8) and a gradient of 8% (v/v) to 100% (v/v) acetonitrile over 18.5 min. A standard curve was generated from a 0.5 M 2-deoxy glucose internal standard. Total AX was calculated from the combined amount of % L-arabinose and % D-xylose, and the molecular weight of two monosaccharides minus one water molecule per monosaccharide (0.88).

### QTL and candidate gene detection

To identify the source of variation in grain AX in the dataset, analysis of variance was carried out using GenStat (15^th^ version). To identify population structure, GenALEx was used to carry out a principal coordinate analysis (PCoA) using data from 104 tetraploid wheat lines, which had been genotyped using the 90K SNP Illumina platform. An unrooted Bayesian tree was constructed using MEGA software version 5.2.2 [24]. The GWAS was carried out in GenStat version 15 using the naïve model and Kinship matrix with SNP map positions [17]. False discovery rate (FDR) <5% was calculated using the q-value package [25] in R version 3.1.1 (R core team 2014) to provide adjusted p values. Some of the markers were genetically mapped using the high-density genetic linkage durum wheat map described by Colasuonno et al. [23] while the rest were located as reported by Wang et al. [17]. From the Wheat Genome Zipper [26], which used homology of the wheat sequences against the annotated proteins in *Brachypodium distachyon*, *Oryza sativa* and *Sorgum bicolor*, the candidate genes involved in AX biosynthesis were identified. Each “gene” or “gene family” name was searched for in the CAZy database [27], in order to find the *Triticum* sequences. All the retrieved protein sequences were BLASTed against the Wheat 61k GeneChip annotated at the PLEXdb database [28], to identify expression data variation.

## Results

### Grain arabinoxylan content

**Fig 1:**
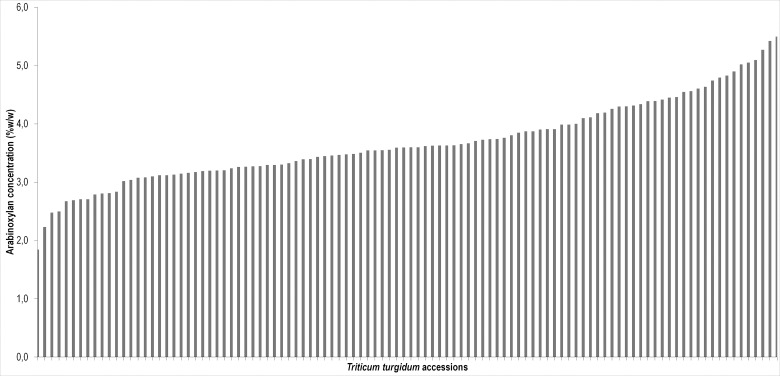
Arabinoxylan content evaluated in a collection of 104 *Triticum turgidum* genotypes. The reported values are the mean of three biological replicates grown at Valenzano 2012.

In order to analyse the genetic variability AX content (AXc) was assessed in a core collection of 104 tetraploid wheat genotypes derived from a collection of 246 accessions grown in Valenzano (Bari, Italy) in 2012. The amount of AX was measured in three biological replicates, and expressed as a percentage w/w of kernel dry weight. AXc ranged from 1.8% to 5.5% (Fig 1) with a mean value of 4.0%. For each sample three technical replicates were analysed and the standard deviation showed non-significant differences between replicates, confirming the reproducibility of the measurement. Analysis of variance showed significant variation (P≤0.001) among the genotypes, hereditability was estimated of 62%. To evaluate the variability among the seven subspecies, into which the collection was divided, the mean values of the subgroups were compared and statistical differences (P≤0.05) were detected. The *durum* accessions showed the highest contents of the collection (5.5%) with mean values ranging from 3.5% to 3.9%. The lowest value was detected in the ssp. *dicoccum* (emmer wheat) with 1.8–3.2% of AX. All other accessions had monosaccharide contents ranging from 3.5% to 4.0% (Table 1).

**Table 1:** Descriptive statistics of AX content in a collection of tetraploid genotypes. Mean values, standard deviation, and minimum and maximum values for grain arabinoxylan content in the *Triticum turgidum* collection. The first row contains the values for the entire collection. All the other data indicates the numbers relative to each subspecies included in the collection.

Sub-species	Number of	Year of	Arabinoxylan		
	individuals	release	Mean	SD	Min-Max
Whole collection			4.00	0.47	1.84–5.50
subsp. *durum*	24	1991–2008	3.57^b.c^	0.55	3.04–5.27
subsp. *durum*	23	1971–1990	3.93^c^	0.72	2.81–5.50
subsp. *durum*	10	before 1970	3.46^b^	0.89	2.52–5.49
subsp. *turanicum*	8		4.01^c^	0.67	3.02–4.83
subsp. *polonicum*	8		4.02^c^	0.70	3.30–5.05
subsp. *turgidum*	12		3.78^b.c^	0.46	3.24–4.74
subsp. *carthlicum*	6		4.11^c^	0.51	3.43–4.64
subsp. *dicoccum*	9		2.59^a^	0.38	1.84–3.17
subsp. *dicoccoides*	8		3.59^b.c^	0.74	2.48–5.09

Different superscripted letters indicate statistically significant differences in arabinoxylan content mean (p ˂ 0.05)

### Molecular data analysis and population structure

The 104 genotypes were analysed using the 90K iSelect array [17]. Out of 81,587 SNP markers, 45,372 (55.6%) were monomorphic and 6346 (7.7%) failed to amplify. We also excluded all the markers located on the D genome (6,519) from the analysis leaving 30294 (37.1%) markers for subsequent analysis. From this subset more SNPs were located on the B genome (16,629) than the A genome (13,665). From the dataset, all the SNPs with allele frequency of >0.95 were deleted providing a total of 22,106 (27%) from the original arrays which satisfied the criteria and were used for the genome-wide association mapping. The highest number of SNPs mapped to chromosome group 1 (3,709 or 16.7%), while group 4 chromosomes had the lowest number of mapped SNPs (2,296 or 10.4%).

**Fig 2:**
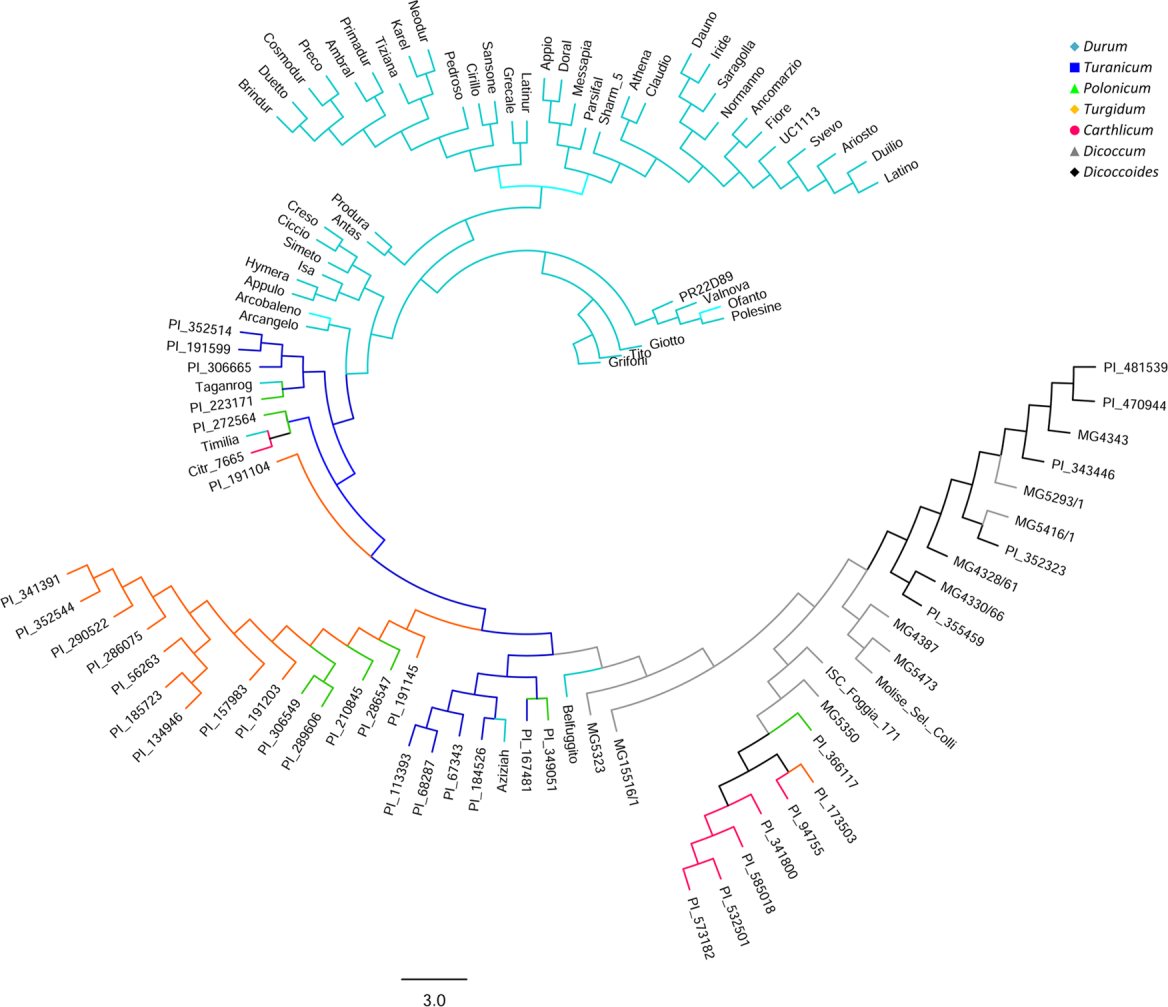
Unrooted Bayesian tree based on 22,106 SNPs spread across the wheat A and B genomes. The tree was constructed from Maximum Likelihood and Bayesian Inference algorithms. Bootstrap values are a percentage of 1000 replicates.

A principal coordinate analysis (PCoA) (data not shown) was carried out with the 104 genotypes and 22,106 markers in order to identify relationships among the subspecies. The first three coordinates explained 28.7%, 9.5% and 7.4% of the variance, respectively for a total of 45.6%. An unrooted Bayesian tree confirmed the groups identified in the PCoA, showing the *durum* accessions clustering together, except for four cultivars (Timilia, Belfuggito, Aziziah, and Taganrog) (Fig 2). The collection was analysed with the Kinship matrix to account for the population structure.

### Analysis of marker-trait associations and QTL detection

**Fig 3:**
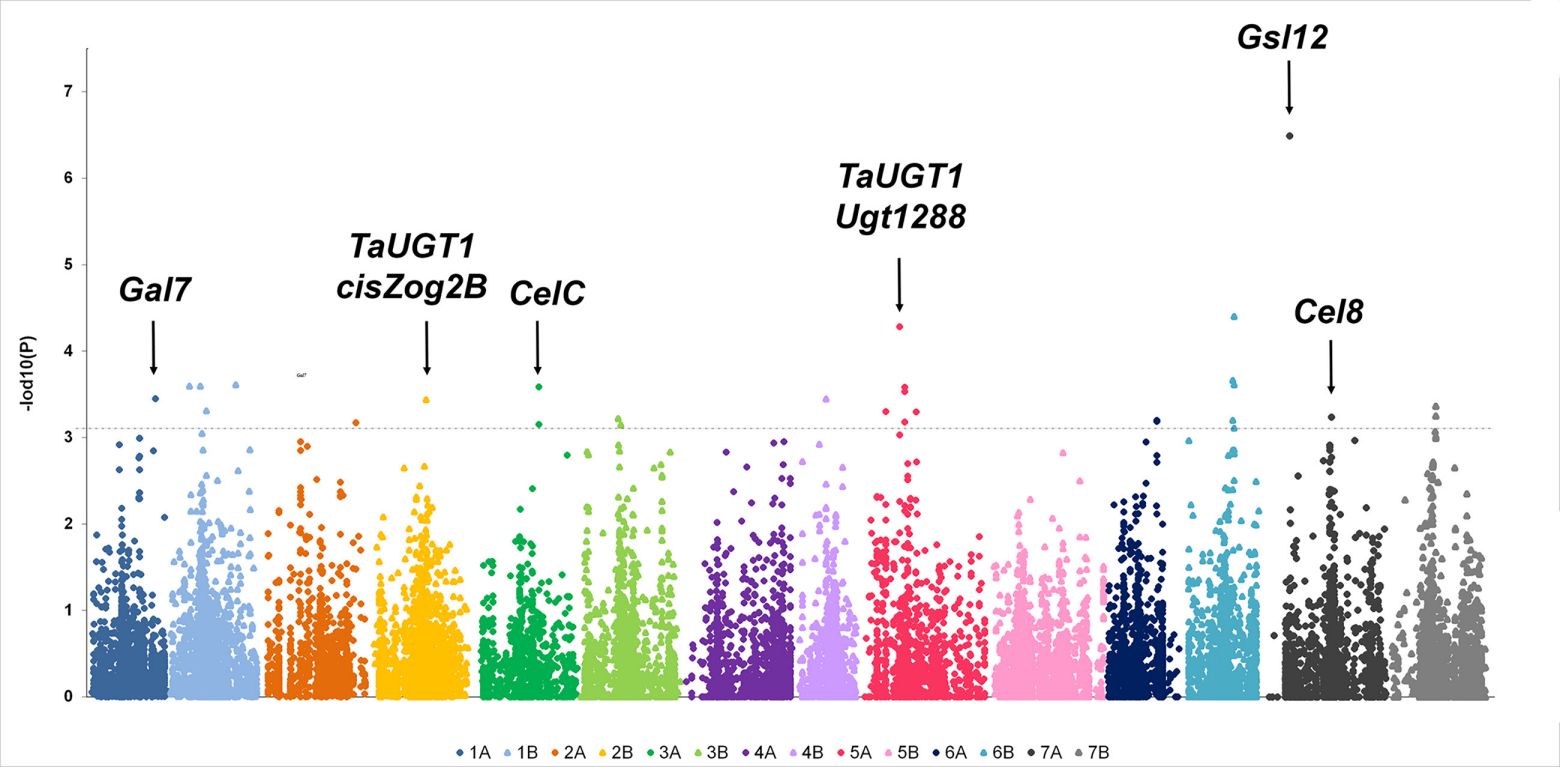
Manhattan plot of grain AX content from GWAS using the Kinship relationship model. The-log10 (p-values) from the GWAS are plotted according to genetic position on each of the 7 wheat chromosome pairs. Positions of candidate genes for AX content are indicated by black downward arrows.

**Fig 4:**
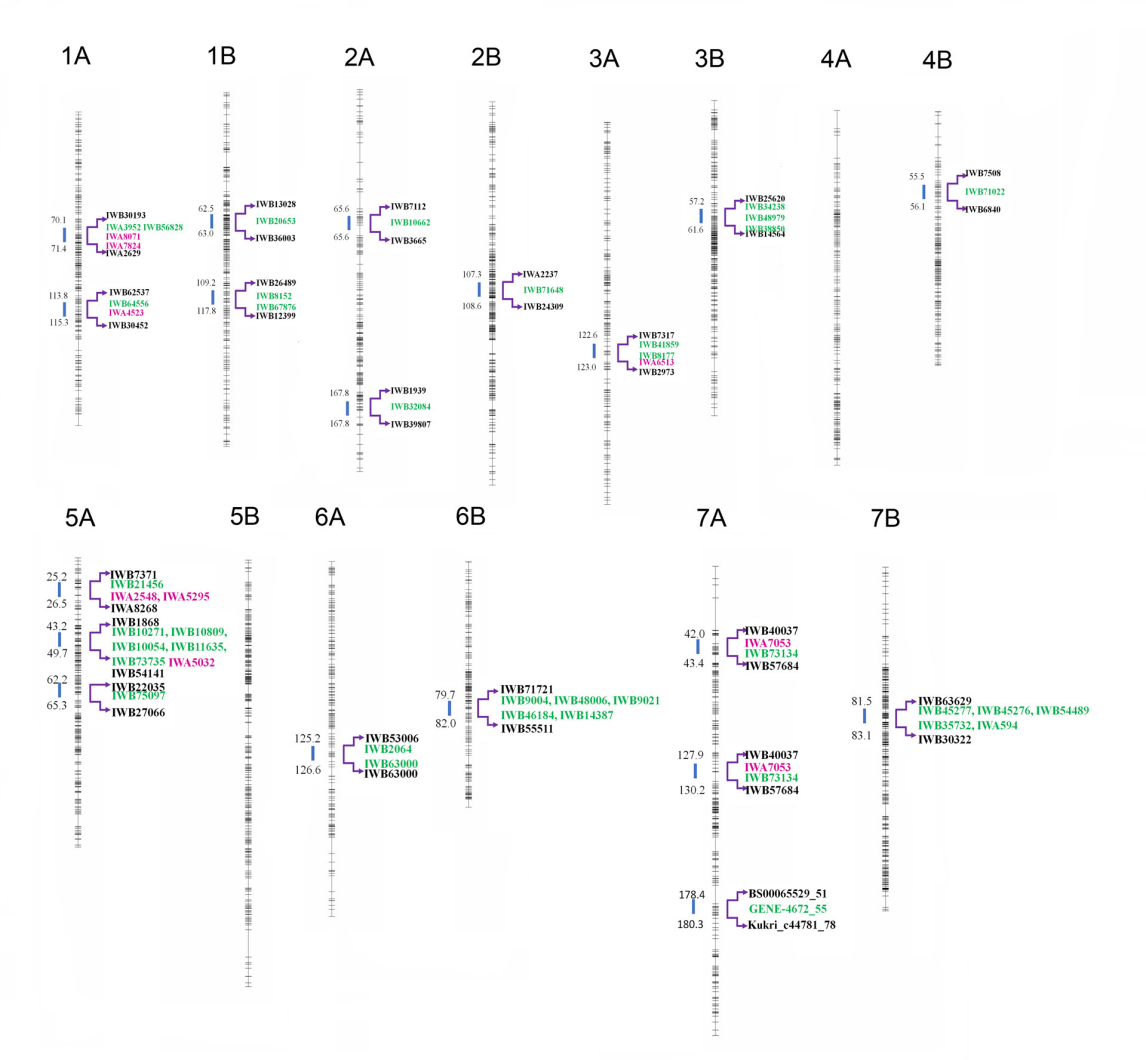
Schematic representation of the seven tetraploid wheat homoeologous chromosome groups analyzed for genome wide association scan. The seven tetraploid wheat homoeologous chromosome groups are characterized by the 22,106 markers used for the genome wide association scan. Chromosome scales are based on the marker position reported by Wang et al. (2014) and each figure was obtained using GenStat 15^th^ edition. On the left side of each chromosome, the putative QTL (indicated by the blue arrows) are reported. The first and the last marker of the QTL region (in black), SNPs with the LOD score ≥3 (in green), and SNPs located in gene sequences encoding AX biosynthesis enzymes (in pink) are indicated on the right hand side.

Associations were calculated using the software package Genstat (15^th^ edition) and individual significant (-Log10 (P) > three) associations between SNP-loci and AX content were detected using a mixed linear model with kinship matrix. A kinship adjusted Manhattan plot summarising the analysis of AX content is shown in Fig 3, and the corresponding naïve analysis in S1 Fig. A-log10 (P) score of 3.0 was taken as the threshold for determining linkage between a marker and the trait (MTA’s). In total 37 MTA’s were detected, identifying 19 regions associated with AX content (Table 2). QTL involved in the accumulation of AX in seeds were found on all wheat chromosomes except chromosomes 4A and 5B. In Fig 4, details are provided of all seven wheat homoeologous chromosome groups with the 22,106 markers used for the GWAS, the 19 QTL, the SNPs with P≥3.0 and their flanking markers within a maximum range of 1.5 cM. The highest number of MTAs identified was on chromosome 5A (seven MTAs identifying three QTL regions), followed by chromosome 7B (five MTAs identifying one QTL region) and chromosome 2A (ten MTAs for two QTL), with the lowest number on chromosome 4B (three MTAs for one QTL) and chromosome 6 (five MTAs for one QTL). With respect to the homoeologous groups, group 7 contained the highest number of MTAs (eight, identifying four QTL), with the lowest numbers for group 4, where only one QTL was detected with one MTA. Altogether, the A-genome had a total of 19 MTAs, for 12 QTL, and the B-genome 18 MTAs, for seven QTL.

**Table 2:** Marker trait association. Associated markers (with-Log10(P) ≥3) QTL for AX content detected by GWAS, using the Kinship matrix. Chromosome location and map position from Wang et al. (2014), -Log10(P) and SNP effect are reported for each SNP.

N°	QTL ID	Closest marker	SNP ID	Chr.	cM	-Log10(P)	Effect
1	QGax.mgb-1A.1	wsnp_Ex_c45880_51550172	IWA3952	1A	70.10	3.06	0.16
2	QGax.mgb-1A.2	RFL_Contig399_976	IWB64556	1A	113.81	3.00	-0.09
3	QGax.mgb-1B.1	Ex_c40520_1484	IWB20653	1B	62.58	3.03	-0.03
4	QGax.mgb-1B.2	BS00039135_51	IWB8152	1B	110.16	3.06	-0.36
5	QGax.mgb-2A.1	BS00073381_51	IWB10662	2A	65.65	3.07	0.39
6	QGax.mgb-2A.2	GENE-0762_808	IWB32084	2A	167.87	3.02	0.31
7	QGax.mgb-2B.1	Tdurum_contig45838_263	IWB71648	2B	107.39	3.04	0.06
8	QGax.mgb-3A.1	Kukri_c17966_634	IWB41859	3A	122.68	3.06	-0.31
9	QGax.mgb-3B.1	GENE-4918_283	IWB34238	3B	57.24	3.02	-0.05
10	Qgax.mgb-4B.1	Tdurum_contig42229_113	IWB71022	4B	55.55	3.04	0.29
11	QGax.mgb-5A.1	Ex_c95453_1499	IWB21456	5A	26.51	3.03	-0.28
12	QGax.mgb-5A.2	BS00068254_51	IWB10271	5A	43.27	4.03	0.48
13	QGax.mgb-5A.3	tplb0056b09_1000	IWB75097	5A	63.69	3.03	-0.38
14	QGax.mgb-6A.1	BobWhite_c27145_318	IWB2064	6A	126.17	3.02	-0.10
15	QGax.mgb-6B.1	BS00063217_51	IWB9021	6B	82.56	4.04	0.37
16	QGax.mgb-7A.1	Tdurum_contig69003_459	IWB73134	7A	42.08	6.05	-0.11
17	QGax.mgb-7A.2	wsnp_Ex_c21854_31021668	IWA2658	7A	130.27	3.02	-0.33
18	QGax.mgb-7A.3	GENE-4672_55	IWB34095	7A	179.24	3.00	-0.32
19	QGax.mgb-7B.1	Kukri_c42653_179	IWB45276	7B	83.07	3.04	0.35

### Candidate gene identification

The 90K Illumina SNP array provided a chromosome marker coverage of the wheat genome of around 85%. Regions of homeologous groups 1, 2, 3, 5, and 7 had significant associations genes known to be involved in the AX biosynthetic pathway (Table 3, Fig 3). For some SNPs the type of change was a synonymous mutation (i.e. wsnp_Ex_c45880_51550172, RFL_Contig399_976, wsnp_Ex_rep_c68194_66973531, Ex_c95453_1499, BS00089966_51, Tdurum_contig69003_459) with no effect on the genomic sequences. S1 Table showed all the markers with non-synonymous change in the sequence determining amino acid substitutions. The available sequences for SNPs reported by Wang et al. [17] were used as queries to run a BLAST search against the wheat GenomeZipper database [26] to identify locations in coding and non-coding regions, their syntenic relationship with *Brachypodium distachyon*, *Oryza sativa* and *Sorgum bicolor* (Table 3), and the putative functions of the encoded proteins. Nine of the 19 markers found to be associated with QTL for grain AX content showed a high sequence similarity with annotated genes encoding enzymes that have been implicated in AX biosynthesis. These include a glycosyl hydrolase from family GH35 [27,29], which encodes *Gal7*, and a glucosyltransferase GT31 on chromosome 1A. There was also a cluster of GT1 genes on chromosome 2B, which included *TaUGT1* and *cisZog1* genes, and a glycosyl hydrolase gene encoding *CelC* on chromosome 3A. Additional genes within regions identified by this analysis included *Ugt12887* and *TaUGT1* on chromosome 5A, a (1,3)-β-D-glucan synthase (*Gsl12*) gene and a glucosyl hydrolase (*Cel8* gene) on chromosome 7A.

**Table 3:** SNP markers used for gene discovery in the AX biosynthetic pathway. The wheat GenomeZipper database was scored for the syntenic relationships between *Triticum* and *Brachypodium distachyon*, *Oryza sativa* and *Sorghum bicolor* sequences. The Carbohydrate-Active enZYmes (CAZy) Database was used to identify the enzyme families and the wheat gene annotations are reported.

N°	Chr	QTL	SNP name	SNP id	cM	Gene annotation in *Triticum* spp.	*Brachypodium distachyon* sequence id	*Oryza sativa* sequence id	*Sorghum bicolor* sequence id	Candidate enzyme	CAZy database
1	1A	QGax.mgb1A.1	wsnp_Ra_c7847_13421750	IWA8071	70.10	-	Bradi2g32570.1	-	-	Glycosyl hydrolase	GH47
			wsnp_Ra_c2895_5488879	IWA7824	71.48	*Gal7*	Bradi2g24670.1	Os05g0428100	-	Glycosyl hydrolase	GH35
2	1A	QGax.mgb1A.2	wsnp_Ex_c6488_11266589	IWA4523	113.77	-	Bradi2g17360.1	Os05g0552200	-	Glycosyltransferase	GT31
3	2B	QGax.mgb2B.1	wsnp_Ex_rep_c68194_66973531	IWA5513	107.01	*TaUGT1*	Bradi5g18010.1 Bradi5g18020.1 Bradi5g18030.1	-	-	Glucuronosyltransferase	GT1
						*cisZog2B*	Bradi5g18040.1	Os04g0565400 Os04g0556500 Os04g0556600	Sb06g024930.1 Sb06g024946.1 Sb06g024960.1 Sb06g024970.1	Cis-zeatin O-glucosyltransferase 1	GT1
	2B		wsnp_RFL_Contig3522_3685860	IWA8478	108.61	-	Bradi5g19840.1	Os04g0589600	-	Glycosyltransferase	GT4
4	3A	QGax.mgb3A.1	wsnp_Ku_c14082_22272647	IWA6513	123.01	*CelC*	Bradi2g59650.1 Bradi2g59660.1	Os01g0930800	Sb03g037780.1	Glycosyl hydrolase	GH1
5	5A	QGax.mgb5A.1	wsnp_Ex_c20611_29693561	IWA2548	26.51	-	-	Os12g0578500	Sb08g019260.1	Glycosyltransferase	GT8
			wsnp_Ex_rep_c66733_65077608	IWA5295	26.51	*Ugt12887*	-	Os12g0583000	Sb08g019540.1	Glycosyltransferase	GT1
6	5A	QGax.mgb5A.2	wsnp_Ex_rep_c101757_87064771	IWA5032	49.23	-	Bradi4g30540.1	-	Sb02g025020.1	Cellulose synthase	GT2
						-	Bradi4g30270.1	-	-	Pectin MethylTransferase	CE8
						-	-	Os09g0416200	Sb02g024690.1	Glucose transporter	-
7	5A	QGax.mgb5A.3	wsnp_Ex_c5626_9897389	IWA4299	62.72	-	-	Os09g0516600	Sb02g029900.1	Glyoxalase II	-
						-	-	Os09g0520200	-	Alpha/beta hydrolase	-
						*TaUGT1*	-	Os09g0518000	-	Glucuronosyltransferase	GT1
						-	-	Os09g0518700	-	Glucosyltransferase	GT1
						-	-	-	Sb02g030090.1	Inositol 1, 3, 4-trisphosphate 56-kinase	-
8	7A	QGax.mgb7A.1	wsnp_Ku_c4299_7814936	IWA7053	42.08	*Gsl12*	Bradi1g51757.1	Os06g0111200	-	1,3-β-D-glucan synthase	GT2/ GT48
9	7A	QGax.mgb7A.2	wsnp_be500615A_Ta_1_1	IWA334	130.27	*Cel8*	Bradi1g44110.1	-	-	Glycosyl hydrolase	GH9
						*Cel8*	-	Os06g0256900	-	β-1,4-endo glucanase	GH9

**Fig 5:**
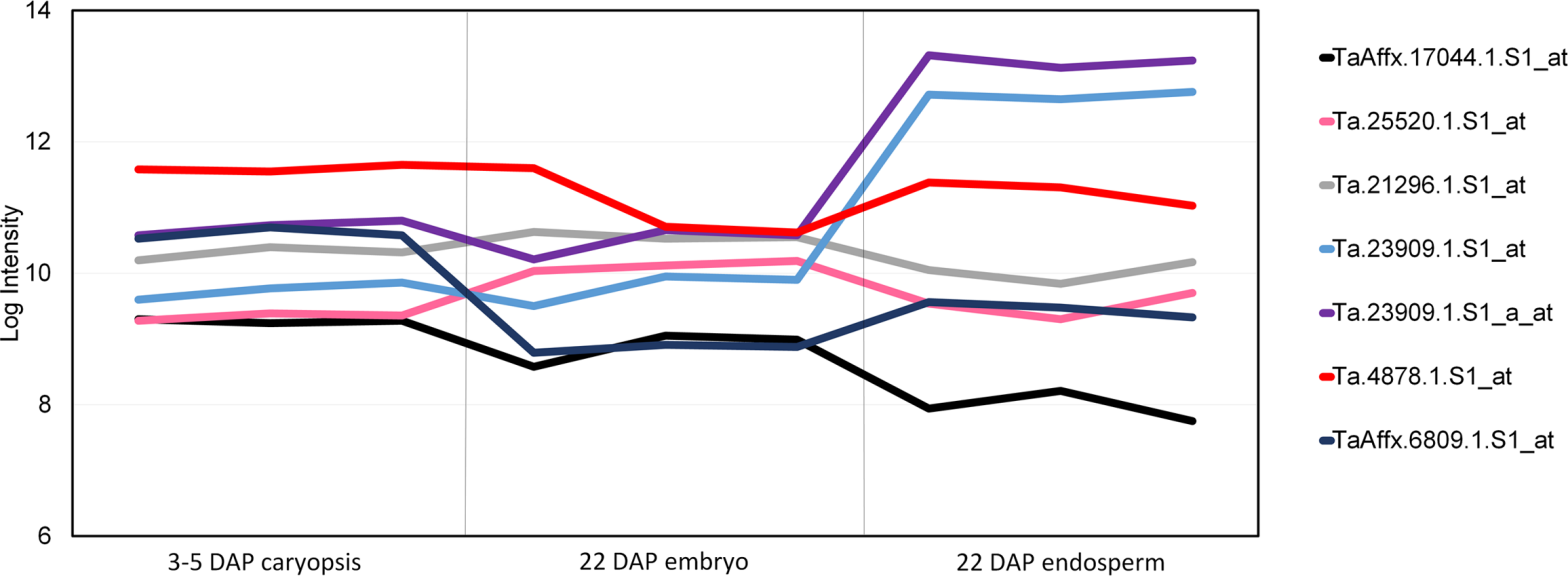
Expression analysis from PLEXdb database of candidate genes for AX biosynthesis. Gene expression measurements are reported on three developmental tissues (caryopsis, embryo and endosperm) for Chinese Spring using the Affymetrix Wheat GeneChip. The Affymetrix code TaAffx.17044.1.S1_at corresponded to the gene *cisZog2B*, Ta.23909.1.S1_at and Ta.23909.1.S1_a_at for *TaUGT1* gene, Ta.25520.1.S1_at and Ta.21296.1.S1_at for *Gsl12*, Ta.4878.1.S1_at and TaAffx.6809.1.S1_at for *Utg12887*.

For all of the candidate genes that have annotations, an *in silico* gene expression analysis was conducted to provide further evidence of their potential involvement in the AX biosynthetic pathway. Exploiting the PLEXdb database [28], the expression data from the Wheat 61k GeneChip was investigated to predict the genes’ impact on the final AX content. In particular, we analysed the transcription pattern variation during different wheat developmental stages (Experiment TA3), focusing on the transcript level in caryopsis at 3–5 days after pollination (DAP), embryos at 22 DAP, and endosperm at 22 DAP (Fig 5). In total seven of the gene sequences showed log intensity values higher than 8 (with RMA normalization), and these corresponded to the queries associated with *TaUGT1*, *cisZog2B*, *Utg12887*, *Gsl12*, all of which encode glycosyl transferase enzymes [27]. The data support the hypothesis of correlation between the transcript levels and the AX content, in fact, abundant transcripts encoding synthetic enzymes give the success in identifying candidate genes for cell wall synthesis from transcriptomics.

## Discussion and Conclusions

Grain dietary fibre content has been shown not only to have important effects on end-use products, on technological properties including milling, baking and animal feed, but also to have significant benefits for human health. AX is the most abundant non-starch polysaccharide constituent in the wheat kernel. In the present study, we report the results of a GWAS of the genetic diversity for AX content in a panel of tetraploid wheat accessions (104 inbred lines). Studies on advanced breeding material could be severely limited by the effects of inbreeding and/or selection. With our approach, using landraces and old cultivars from distinct geographical origins, and having a longer history of recombination events, we dilute the LD effects related to inbreeding. GWAS has proven to be a powerful tool to dissect quantitative traits. This is because the technique potentially provides higher resolution than traditional QTL mapping. This, combined with increasing information from annotated genome sequences [26] and genetic resources such as gene expression datasets [28], allows improved prediction of candidate genes from the regions indicated as being of interest from the GWAS. The population structure analysis carried out Laido et al. [19] on a collection of *Triticum turgidum* genotypes from which the germplasm described in the current study are a subset, indicated that a degree of population structure could be detected and that the results from this analysis should be interpreted with caution. In the present report for the first time a the new class of markers, single-nucleotide polymorphism (SNP) useful in the development and saturation of genetic maps was applied for the detection of QTL regions involved in the control of arabinoxylan content in tetraploid wheat.

GWAS conducted on a set of 104 tetraploid wheat genotypes identified several chromosomal regions correlated with the AX content of grain. A total of 37 marker-trait associations were detected, identifying 19 QTL regions. The polygenic, quantitative nature of this trait was confirmed by the presence of putative QTL on all chromosomes except 4A and 5B. Four QTL were detected on chromosomes 1A and 1B in homoeologous positions and on the homoeologous group 7 chromosomes, three QTL on groups 2 and 5, two QTL on homoeologous groups 3 and 6 and one QTL was found on homoeologous group 4. In a previous study, Nguyen reported QTL associated with AX content on chromosomes 1A, 2A, 6B, and 7A [13]. Although only a chromosome comparison is possible as this is the first QTL map obtained with SNP markers, looking at the map position we suppose a coincidence of QGax.mgb-1A.1, QGax.mgb-2A.1, QGax.mgb-6B.1 and QGax.mgb-7A.2. The presence of a major QTL located on chromosome 1B was detected by Martinant et al. [10]. Recently a MetaQTL analysis for fibre content [14] found loci for AX content on wheat chromosomes 1B, 3A, 5B, 6B, 7A, 7B. Here, we report for the first time QTL for AX content on chromosomes 2B and 5A, including three important regions on chromosome 5A that are strongly associated with AX content.

The AX values obtained in our core collection ranged from 1.8% to 5.5%, with a mean value of 4.0%, the hereditability was of 62% value comparable with that reported by Shewry et al. [30]. While the upper range of this dataset is comparable to the AX content described by Ciccoritti et al. [11] and Lempereur et al. [31], who reported a range from 4.1% to 5.8% and from 4.07% to 6.02% respectively, the current study identified a larger range of AX values. This is likely to be a reflection of the fact that the current study sourced material from a wider range of accessions, whereas Cicoritti et al. [20] focused on *Triticum turgidum L*. *var durum*.

Our study detected nine QTL on chromosomes 1A, 2B, 3A, 5A, and 7A in regions coding for genes for AX biosynthetic pathway enzymes. These candidates include glycosyl transferase (GT) and glycosyl hydrolase (GH) enzymes which were previously found from QTL analyses for AX content [12–14]. More precisely, the GT candidates identified included a GT1 cluster on chromosome 2B, which includes TaUGT1 and cisZog1, a GT1 cluster on chromosome 5A, which includes *TaUGT1* and *Ugt12887*, and a *GT48* gene. Previous studies indicated groups of genes in the families’ CslC, GT2, GT43, GT47, GT48, GT61, GT64 or GT77 as potential candidate genes for AX biosynthesis, with most evidence implicating genes that encode proteins from the GT43, GT47, and GT61 families [6]. We also detected several glycosyl hydrolases, including one GH35 gene (*Gal7*) on chromosome 1A, one GH1 (*CelC*) on chromosome 3A, and two members of the GH9 family (*Gsl12* and *Cel8*) on chromosome 7A. Pellny et al. [32] showed high levels of GH9 transcripts related to known cellulases in the starchy endosperm of wheat. Our analysis did not detect any significant associations with GT43, GT47 or GT61 genes.

In conclusion, this GWAS allowed us to identify new QTL for AX content and has contributed to our understanding of the genetic complexity of this important agronomic trait. It has allowed us to evaluate the variation in AX content in this collection of *Triticum turgidum*, also known as durum wheat, which is an economically important species. New putative candidate genes that could be directly involved in AX synthesis or in the regulation of the process have been identified. This could be of great importance for further genetic studies such as validation of the function of these candidate genes in the AX biosynthetic pathway, resulting in a more efficient use of genetic resources in breeding programs to obtain more productive and adaptable varieties.

## Supporting Information

S1 Fig Manhattan plots of grain AX content GWAS using the naïve model. The-log10 (p-values) from a genome-wide scan are plotted against the position on each of the 7 wheat chromosome pairs.
(PDF)

S1 Table Marker trait association with non-synonymous change in the sequence. (PDF)
