# Supplementary figures and images for: Genome Wide Association Mapping for Arabinoxylan Content in a Collection of Tetraploid Wheats
Source: PLoS One. 2015 Jul 15;10(7):e0132787. doi: 10.1371/journal.pone.0132787 (PMC4503733; doi:10.1371/journal.pone.0132787)

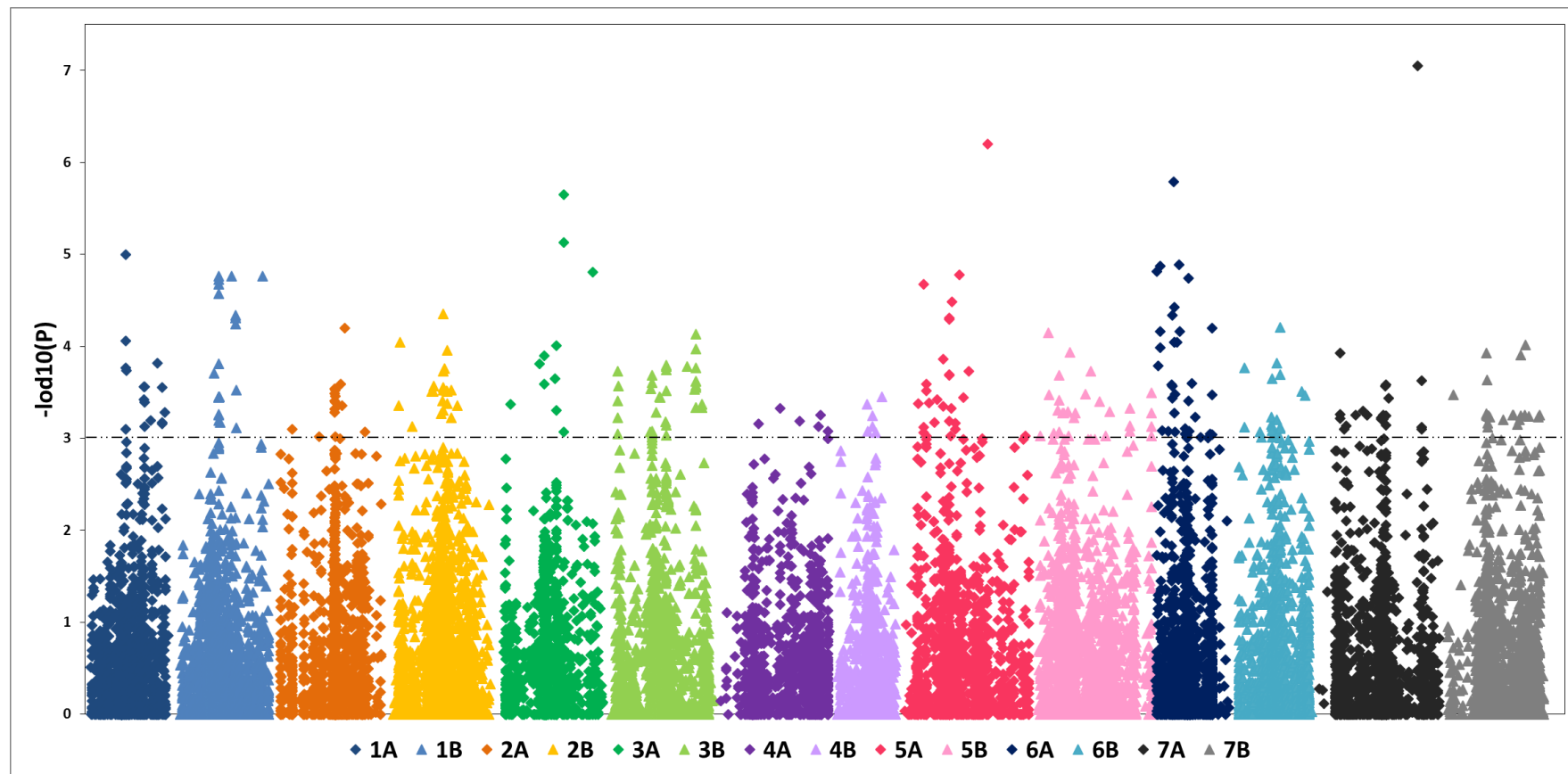

S1 Fig

Supplement: S1 Fig — The-log10 (p-values) from a genome-wide scan are plotted against the position on each of the 7 wheat chromosome pairs. (PDF) [file pone.0132787.s001.pdf]
