# Supplementary material for: Genome Wide Association Mapping for Arabinoxylan Content in a Collection of Tetraploid Wheats
Source: PLoS One. 2015 Jul 15;10(7):e0132787. doi: 10.1371/journal.pone.0132787 (PMC4503733; doi:10.1371/journal.pone.0132787)
Supplement: S1 Table — (PDF) [file pone.0132787.s002.pdf]

S1 Table

SNP ID	Mutation	Amino Acid Change	Type of change
IWA8478	[T/C]	_->Q	Transition
IWB41859	[T/C]	I->V	Transition
IWA2548	[A/G]	S->P	Transition
IWB10271	[A/G]	I->T	Transition
IWA5032	[T/C]	R->G	Transition
IWA7053	[A/G]	I->T	Transition
IWA2658	[T/C]	L->S	Transition
